# Placing Trust at the Heart of Health Policy and Systems

**DOI:** 10.34172/ijhpm.2024.8410

**Published:** 2024-05-07

**Authors:** Martin McKee, May CI van Schalkwyk, Rachel Greenley, Govin Permanand

**Affiliations:** ^1^Department of Health Services Research and Policy, London School of Hygiene & Tropical Medicine, London, UK.; ^2^Division of Country Health Policies and Systems, World Health Regional Office for Europe, Copenhagen, Denmark.

**Keywords:** Trust, Health Systems, Resilience, Patient-Centredness

## Abstract

Trusted interactions are crucial in health systems. Trust facilitates effective healthcare by encouraging patients to seek and adhere to treatment, enabling teamwork among health professionals, reducing miscommunication and medical errors, and fostering innovation and resilience. The COVID-19 pandemic underscored the importance of trust, highlighting the challenges in establishing and maintaining it, especially during crises when trust in authorities and health systems is vital for compliance and safety. However, trust is complex, varying with context and experiences, and is dynamic, easily lost but hard to regain. Despite its importance, trust is often overlooked in health policy and difficult to measure. Health systems and policy-makers must recognize the importance of trust, measure it effectively, understand how it is built or eroded, and act to maintain and restore it. This involves acknowledging the past experiences of marginalized groups, involving communities in decision-making, and ensuring transparency and integrity in health practices and policies.

## Why Are Trusted Interactions Important?

 Health systems involve a series of interactions between different people. These include patients who seek care (or who should but do not or cannot) and the health workers who provide it. They include those who manage health systems and produce the resources necessary for them to function, whether as educators training a new generation of health workers, or scientists and manufacturers developing and delivering what they need, now increasingly complex with the emergence of biological therapeutics and the algorithms that power artificial intelligence (AI). They include those that make the policies within which health systems operate, such as politicians, insurance funds and private corporations.

 Like all human interactions, these will be most effective where they are based on trust. Trust encourages people to seek care when in need and to adhere to treatment.^1^ It helps health professionals to work together in teams,^2^ essential if they are to deliver multidisciplinary care. It reduces the potential for miscommunication and, thus, medical errors. It increases the sense of well-being within these teams, as members know that their colleagues will support them,^3^ and it supports innovation where individuals feel secure in taking calculated risks to drive improvements in healthcare.^4^ It enables faster decision-making as it reduces the inclination to question the motivations of others. Finally, it promotes resilience in times of crisis.^5^ But do health systems prioritise building and maintaining trust? In this editorial, which draws on a much longer report on trust and transformation written for a 2023 World Health Organization (WHO) meeting that also includes an extensive review of different disciplinary perspectives on trust, we argue that they rarely do and explain why this needs to change.^6^

## Lessons From the Pandemic

 The importance of trust became apparent during the COVID-19 pandemic.^7^ Faced with a new virus about which, at least initially, there was much uncertainty, populations around the world were called upon to place their trust in authorities, especially politicians and the scientists advising them, who asked them to make previously unimaginable changes to their behaviour.^8^ Health workers, some of whom would die after contracting COVID-19, had to trust that these authorities were doing what they could to protect them. Those who might need care in a hospital or care home, many of whom would also die after contracting COVID-19 in those facilities, had to trust that everything possible was being done to limit the spread of the virus in facilities that could all too easily become incubators of infection.

 The challenges involved in creating these trusted relationships were enormous. While many people were doing what they could to build trust, others adopted practices that actively undermined it. Those complicit in undermining trust included individuals who sought to exploit the crisis, profiteering from the shortages of supplies brought about by governments that had failed to plan.^9^ They also included those who, for a variety of motives, were spreading disinformation, undermining trust in the restrictions necessary to reduce the spread of infection or the vaccines that had been developed by dedicated teams of researchers working tirelessly and at unprecedented pace.^10^

 Yet, it is not only at times of crisis that a loss of trust is problematic. Solomon and Flores explain that trust must be built and maintained actively and continually, involving an interplay of ethics, risk, vulnerability, and communication.^11^ Furthermore, there are different forms and depths of trust, including basic intuitive and norms-based initial trust to the complexity of authentic trust that acknowledges the potential for betrayal yet chooses to trust nonetheless. This work speaks more to the paradox of cordial hypocrisy,^12^ where outward appearances of trust mask underlying distrust, highlighting the role of social and political mechanisms to maintain civility. Drawing on this nuanced conceptualization of trust, we have identified three main sets of relationships where trust is crucial.^13^ One is the simple trust that patients and the public have in health and care systems, essential for securing their support for the principle of solidarity, which is a commitment within a group to mutual support and shared responsibilities,^14^ that underpins effective systems and ensuring that they seek and accept care without delay when they need it. This trust is often based on past experiences or future expectations, although it is vulnerable to experiences of betrayal or disappointment. A second is the basic trust between health workers and their employers. This trust has a default expectation that, on a fundamental level, things are reliable and that others generally have good intentions, without which workers will seek other careers or move abroad. A third is the trust that politicians must have in health systems if they are to commit funds to those systems in the belief that their investment will bring improvements. The extent of this trust may vary, depending on certain socio-political contextual underpinnings. To this can be added many others, such as the trust by politicians that the public would do as advised in a crisis such as the pandemic or whether punitive sanctions, such as fines, would be required to make them comply,^15^ or the trust of health workers in the safety of the technology or medicines they use in their practice. The latter requires trust in both the manufacturers and those who regulate them, something that is being challenged by the expansion of AI where clinicians must trust the technology enough to use it but not so much that they surrender their clinical judgement inappropriately.^16^

 Yet, although it is obvious that trust is essential in any well-functioning health system, it is not something we can take for granted, and especially not given experiences during the COVID-19 pandemic. As the pandemic unfolded, it systematically disrupted the foundational elements of trust within society. The once-unquestioned simple trust was eroded by the flow of constant uncertainty and changing health advice. Basic trust in established institutions experienced changed, as pandemic preparedness and response measures, such as social distancing or vaccination, became contested. Authentic trust faced the ultimate test, challenged by a sense of helplessness in the face of shared experiences of illness and adversity during the pandemic. Finally, we saw cordial hypocrisy increased, serving as a social placebo, preserving a façade of cohesion against a backdrop of collective threat. It is not inevitable that health professions or the health system will command public trust and nor should they be seen as inherently trustworthy.

## Measuring Trust

 Despite its centrality, it is remarkable how little attention trust receives in health policy. It is largely absent from the metrics that are used to evaluate health system performance. It is discussed when things go wrong, emerging as a frequent factor in reports into clinical failures but is soon forgotten about, displaced by organisational or regulatory change that can make things worse. So why is this the case?

 An obvious problem is that trust is complex. There are many ways of looking at it, it can take many different forms, there are marked differences in how people interpret the concept and questions used to elucidate aspects of trust, and our report on which this editorial draws describes how there is little evidence of synthesis of ideas from the main disciplines that have examined it, such as philosophy, psychology, sociology, and economics.^6^ Although much is known about trust, this knowledge exists largely in silos that are isolated from each other. The situation is further complicated by the way in which the meaning of “trust” and related words vary in different languages.^17^ Trust is also dynamic. It can take years to build or restore trust, while it can be eroded in a matter of minutes or even seconds in a digitally connected world. A Dutch proverb captures this well: “*Trust arrives on a tortoise and leaves on a horse.” *Furthermore, trust is not inevitably all-encompassing and can be confined to a specific relationship or competency. For example, someone may trust their general practitioner to maintain patient confidentiality and help in the management of diabetes care but would not trust them to perform major cardiothoracic surgery competently and safely. A doctor may trust AI to identify the presence of signs of pneumonia on a chest X-ray but not accurately stage a patient’s lung cancer and develop a chemotherapy regime.

 Given all these considerations, it is perhaps unsurprising that there is no single, universally recognised definition of trust even though, as is the case for many terms in widespread use, like love or affection, we often know whether it is there or not. Russell Hardin, a leading scholar on trust noted that “*To say we trust you means we believe you have the right intentions towards us and that you are competent to do what we trust you to do.”*^18^ Unfortunately, while this is easy to say it is difficult to measure. It cannot be quantified as easily as other elements of the health system like levels of funding or numbers of health workers. It pushes the boundaries of how we measure and monitor health systems and their constituent elements. However, as John Clifton, Chief Executive Officer of Gallup has argued, the same is true of many other important constructs that pollsters have succeeded in measuring.^19^

 Efforts to measure and track trust will therefore need to reflect this complexity, which at a minimum will require multiple but complementary measures, novel approaches and careful evaluation and validation. Such efforts will also need to take into consideration the fact that trust varies with context, including the experiences of those involved. The level of trust in a health system, or in any part of it, can reflect levels of trust more widely in the societies in which it is embedded, and it is not evenly distributed within those societies. Building trust involves acknowledging people’s collective experiences, past and present, and particularly the experiences of those who have been marginalised and mistreated by the health system and by those delivering public services more generally. To take one example, it is perfectly rational that an undocumented migrant, who has endured horrendous experiences on their journey to a new country and, in many cases after their arrival, should initially lack trust in even the most sympathetic health worker. The same is true for minority populations, defined by their ethnicity, sexuality, or other characteristics, that have experienced discrimination, placing an obligation on clinicians treating them to be aware of their history.^20^ Recognising the multiple and interconnected drivers of people’s mistrust has implications for how trust is measured among individuals and groups as well as for identifying what is needed to (re)built trust and reform social and political institutions so that they are *trustworthy*, emphasising the need for constant reflection on how health systems can remain trustworthy for everyone. A similar logic applies to building trust among healthcare workers, whose trust may be lost for very different reasons including the embedding of blame or litigious cultures within the healthcare sector, which will in turn require different forms of inquiry and actions to (re)build trust.

## Trust and Transformation

 Given these challenges, it is perhaps understandable that health policy-makers have placed trust in the “too difficult” tray. Cynically, some might feel that the politicians making health policy have an additional incentive to do so given evidence from polling that they are often among the least trusted in society. Yet, as the pace of change in healthcare accelerates, with the growth of multimorbidity in ageing populations, new opportunities to intervene, and evidence of the effectiveness of novel models of care, health systems will face increasing pressure to transform themselves. Indeed, if they are to build and retain trust from the public and their political representatives, they must show that they can adapt to the changing needs of patients while anticipating the challenges posed by unfolding crises and novel threats. Effective transformation is thus core to maintaining trust that a health system will be there when needed and provide quality and equitable care now and into the future.^13^

 Yet any change is often unpopular among those affected as it brings risks and disruption. This can only be addressed if there is a vision that everyone affected can trust. This vision must be one that people believe in and see it as a means to protect and promote the public interest. Thus, successful transformation is only possible with trust, and trust can only be earned by involving and listening to those who are being asked to give their trust. Without trust, health systems cannot transform in a positive way, and without transformation that benefits all, they cannot garner trust or remain trustworthy.

 There are many reasons for concern. In some places, the public is losing trust in their health systems’ ability to meet their needs and provide safe care and in the science that underpins it. Health workers are losing trust in the ability of the health system to enable them to meet their aspirations, to realise their potential, and sometimes simply to treat them with respect and allow them to deliver the care they know their patients need. Policy-makers are losing trust that health systems can transform and use public funds effectively in a world that is becoming ever more complex.

## What Needs to Happen?

 This situation cannot continue. Health policy makers and researchers must recognise the importance of trust and take it much more seriously, especially given the experience of the pandemic. This means, firstly, measuring it. There are methodological challenges but there is also a wealth of experience among, for example, political scientists and polling companies.^21,22^ This will, however, require spending money on surveys, ideally using standardised measures to facilitate comparisons and questions that are inclusive of different cultures and relationships. Second, it requires qualitative and cross-disciplinary research that can help understand how trust is eroded and how it can be built, restored, and protected. Third, it requires those in authority to behave in ways that maintain trust and that they remain trustworthy. This is perhaps the most difficult, especially as some politicians have seen their path to victory as being helped by undermining trust, exploiting so-called wedge issues that fuel culture wars. When this happens, it is incumbent on the health community to call it out, rejecting attempts to sow divisions and consequently undermine societal trust. And, of course, health workers themselves must behave in ways that build and maintain trust and ensure the trustworthiness of health system. For example, depending on the context and evidence on what is fuelling a loss of trust, this may involve rooting out conflicts of interest and corruption when it exists in their health systems,^23^ although often this will require concerted political action too. Informal payments, while inexcusable, can be explained by the failure of many governments to pay their staff properly.^24^

 For too long, too many of those involved in health policy, as practitioners or researchers, have overlooked or undervalued the importance of trust. This must change and, as we reflect on the lessons from the pandemic and look at how we will respond to future threats, such as the climate emergency, and how we can design systems that are person-centred and promote a vision of health and well-being that defines those seeking care not in terms of their clinical conditions but as whole persons living lives that are embedded in communities. We can take advantage of a nascent, but growing body of evidence, coupled with opportunities to gather new types of data offered by advances in technology, to reveal where trust is lacking. But measuring is one thing and acting on the knowledge this brings is another. Endeavours to measure and build understanding of trust, trustworthiness and what cultivates or erodes trust will need to be translated into effective actions that rebuild and maintain trust over time and among everyone. Commitments to respect and value trust and its core role in health system functioning and transformation that are not followed by sincere and meaningful actions will only fuel mistrust and undermine the trustworthiness of health systems.

## Acknowledgement

 This editorial is based on work undertaken in preparation for a Ministerial Conference organised by the European Regional Office of WHO on Trust and Transformation, held in Tallinn, Estonia, in December 2023.

## Ethical issues

 Not applicable.

## Competing interests

 The authors are either employees of WHO (GP) or received support from WHO and the Observatory to attend and participate in the WHO conference on which this work is based. MM is (unpaid) Research Director of the Observatory but receives travel expenses and his employer receives administrative and salary support for other staff.

## Funding

 This editorial draws on work undertaken by the authors in the course of their work for WHO Europe or the European Observatory on Health Systems and Policies but no specific funding was required for writing it.
